# It needs more than a myocardial infarction to start exercising: the CoLaus|PsyCoLaus prospective study

**DOI:** 10.1186/s12872-024-03755-9

**Published:** 2024-02-12

**Authors:** François Flammer, Anisoara Paraschiv-Ionescu, Pedro Marques-Vidal

**Affiliations:** 1 Department of Medicine, Internal Medicine, Lausanne University Hospital and University of Lausanne, 46 rue du Bugnon, Lausanne, 1011 Switzerland; 2 Laboratory of Movement Analysis and Measurement (LMAM), Ecole Polytechnique Federale de Lausanne (EPFL), Lausanne, Switzerland

**Keywords:** Physical activity, Secondary prevention, Cardiovascular disease, Prospective study, Switzerland

## Abstract

**Background:**

Increased physical activity (PA) is recommended after an acute coronary event to prevent recurrences. Whether patients with acute coronary event actually increase their PA has not been assessed using objective methods such as accelerometer. We aimed to assess the subjectively and objectively measured physical activity (PA) levels of patients before and after an acute coronary event.

**Methods:**

Data from the three follow-up surveys of a prospective study conducted in Lausanne, Switzerland. Self-reported PA was assessed by questionnaire in the first (2009–2012) and second (2014–2017) follow-ups. Objective PA was assessed by a wrist-worn accelerometer in the second and third (2018–2021) follow-ups. Participants who developed an acute coronary event between each survey period were considered as eligible. PA levels were compared before and after the event, and changes in PA levels were also compared between participants who developed an acute event with three gender and age-matched healthy controls.

**Results:**

For self-reported PA, data from 43 patients (12 women, 64 ± 9 years) were used. No differences were found for all PA levels expressed in minutes/day before and after the event: moderate PA, median and [interquartile range] 167 [104–250] vs. 153 [109–240]; light PA: 151 [77–259] vs. 166 [126–222], and sedentary behaviour: 513 [450–635] vs. 535 [465–642] minutes/day. Comparison with gender- and age-matched healthy controls showed no differences regarding trends in reported PA. For accelerometer-assessed PA, data from 32 patients (16 women, 66 ± 9 years) were used. No differences were found for all PA levels expressed in minutes/day before and after the event: moderate PA: 159 [113–189] vs. 141 [111–189]; light PA: 95.8 [79–113] vs. 95.9 [79–117], and sedentary behaviour: 610 [545–659] vs. 602 [540–624]. Regarding the comparison with gender- and age-matched healthy controls, controls had an increase in accelerometer-assessed sedentary behaviour as % of day: multivariable adjusted average standard error 2.7 ± 0.6, while no increase was found for cases: 0.1 ± 1.1; no differences were found for the other PA levels.

**Conclusion:**

Patients do not seem to change their PA levels after a first coronary event. Our results should be confirmed in larger samples.

**Supplementary Information:**

The online version contains supplementary material available at 10.1186/s12872-024-03755-9.

## Background

Adequate physical activity (PA) reduces the risk of morbidity after an acute myocardial infarction (AMI) [1] or a stroke [2]. Guidelines have been issued regarding the prescription of PA [3, 4], but rehabilitation programs are far from optimal in most European countries [5, 6]. Previous studies reported that people with AMI or cardiovascular disease (CVD) display lower PA levels than healthy counterparts [7], although this statement has been challenged [8]. Exercise-based cardiac rehabilitation has been shown to decrease recurrence and mortality in patients with coronary artery disease [9] Still, a recent study conducted in Korea showed that only 22% of patients who presented a CVD event increased their PA level, and that this change in PA led to a decrease in mortality [10]. Still, most studies relied on self-reported PA or cross-sectional data and, to our knowledge, no study ever objectively assessed PA levels before and after a first coronary event.

Hence, we aimed to assess subjectively and objectively measured PA levels in subjects before and after the occurrence of a coronary event, and to compare the trends in PA metrics in patients with a coronary event with gender- and age-matched controls. Our hypothesis was that subjects who survived a coronary event would increase their PA levels to prevent recurrence.

## Methods

### Sampling

The CoLaus|PsyCoLaus study is a population-based prospective study assessing the clinical, biological, and genetic determinants of cardiovascular disease aged 35 to 75 years at baseline, living in the city of Lausanne, Switzerland [11]. In each survey, participants answered questionnaires, underwent a clinical examination and blood samples were drawn for analyses. Recruitment began in June 2003 and ended in May 2006 and included 6733 participants. The first follow-up was performed between April 2009 and September 2012 and included 5064 of the initial participants. The second follow-up was performed between May 2014 and April 2017 and included 4881 participants. The third follow-up was performed between April 2018 and May 2021 and included 3751 participants. Median follow-up time was 5.4 (average 5.6, range 4.5–8.8) years for the first follow-up, 10.7 (average 10.9, range 8.8–13.6) years for the second follow-up, and 14.5 (average 14.6, range 13.2–17.3) for the third follow-up. For more details, see www.colaus-psycolaus.ch.

PA levels were subjectively assessed between the first and the second examination using a questionnaire validated for the French-speaking Swiss population. PA levels were objectively assessed between the second and the third examination using a wrist-worn accelerometer.

### Self-reported physical activity

The Physical Activity Frequency Questionnaire (PAFQ) has been validated in the population of Geneva, Switzerland [12]. This self-reported questionnaire assesses the type and duration of 70 kinds of (non)professional activities and sports during the previous week. For the purpose of this study, each type of activity was categorized into sedentary behaviour (SB, < 2 metabolic equivalent of tasks – METs), light physical activity (LPA, 2 to < 3 METs), moderate physical activity (MPA, 3–6 METs) and vigorous physical activity (VPA, > 6 METs) according to the compendium of physical activities [13, 14]. For each item of the PAFQ, the time spent per week was computed as average hours per day multiplied by the number of days performing the activity. For each item category (i.e., corresponding to SB, LPA, MPA or VPA), the times were summed up and divided by 7 to estimate an average daily time. For example, if the participant spent 2 h/day housekeeping (MPA) and performed this activity for 3 days per week, then the total time was 2 × 3 = 6 h/week; if the participant also spent 1 h/day sewing and ironing (MPA) and performed this activity every day, then the total time was 1 × 7 = 7 h/week, and the average daily time spent in MPA activities would be (6 + 7)/7 = 1.85 h or 111 min.

Physical activity metrics were extracted as described previously [15]. Usual sleep time (in minutes) was assessed by asking the participants when they went to bed and when they woke up, and the number of minutes on non-sleep time (NST) was computed as NST = 1440–sleep time. As participants tended to under or overestimate time spent in the different activities, a standardization was performed as follows: first, we calculated T, the total amount of time spent in SB, LPA, MPA and VPA activities. Second, we computed the percentage of time dedicated to each type of activity, i.e., PSB = time spent in SB/T. Third, we computed the standardized time spent on each type of activity by multiplying the non-sleep time by the percentage of time spent in each activity STSB = NST×PSB [15]. A detailed example of calculation is provided in Supplementary information.

### Accelerometer-assessed physical activity

Physical activity was objectively assessed using a wrist-worn triaxial accelerometer (GENEActiv, Activinsights Ltd, United Kingdom, www.activinsights.com). These devices are the same that have been used in the UK biobank study [7] weight 16 g, and allow continuous monitoring of physical activity for a maximum of 45 days. The devices were pre-programmed with a 50 Hz sampling frequency and subsequently attached to the participants’ right wrist. Participants were requested to wear the device continuously for 14 days in their free-living conditions.

Raw accelerometer data was analysed using the software GGIR version 1.5-9 running on R [16], using the acceleration thresholds proposed by White et al. [17] to define LPA (≥ 85 and < 181 milli-g), MPA (≥ 181 and < 437 milli-g) and VPA (≥ 437 milli-g). A second extraction of PA metrics was performed using the GENEActiv software version 2.9 (GENEActiv, Activinsights Ltd, United Kingdom) and transformed into 1-minute epoch files. Data were then analysed using the GENEActiv macro file ‘General physical activity’ version 1.9, which had been previously validated [18]. A valid day was defined as ≥ 10 h (i.e. 600 min-epoch) and ≥ 8 h (i.e. 480 min-epoch) of diurnal wear-time on weekdays and weekend days, respectively. Walking metrics were extracted from the raw accelerometer data using a validated algorithm [19, 20]. Mean number of steps per day for walking bouts of at least 30 or 120 s, mean duration of walking bouts, and mean walking cadence (number of steps per minute) were extracted from the raw accelerometer data.

### Outcomes

Acute myocardial events were adjudicated by two cardiologists, and stroke by one neurologist; all specialists were external to the CoLaus|PsyCoLaus study staff [21]. Outcomes were defined according to Goff et al. [22, 23]. A coronary event was defined as angina pectoris, unstable angina, myocardial infarction, or coronary revascularization (angioplasty or CABG). Stroke was not included as it could impact mobility and thus PA levels. The detailed adjudication procedure is provided in the Supplementary information.

### Covariates

The collection and characterization of most covariates has been provided previously [11]. Smoking status was self-reported and categorized as never, former (irrespective of the time since quitting) and current [11]. Educational level was categorized as high (university), middle (high school) and low (mandatory or apprenticeship). Body weight and height were measured with participants barefoot and in light indoor clothes. Body weight was measured in kilograms to the nearest 100 g using a Seca® scale (Hamburg, Germany). Height was measured to the nearest 5 mm using a Seca® (Hamburg, Germany) height gauge. Body mass index (BMI) was computed and further categorized as normal (< 25 kg/m^2^), overweight (25-29.9 kg/m^2^) and obese (≥ 30 kg/m^2^) [11]. Blood pressure (BP) was measured using an Omron® HEM-907 automated oscillometric sphygmomanometer after at least a 10-minute rest in a seated position, and the average of the last two measurements was used. Hypertension was defined by a SBP ≥ 140 mm Hg or a DBP ≥ 90 mm Hg or presence of antihypertensive drug treatment [11].

### Inclusion and exclusion criteria

Participants were included if they (1) presented with a first coronary event during the follow-up period and (2) had either subjective or objective PA data before and after the outcome. Participants were excluded if they (1) did not attend the examination after the outcome (no follow-up); (2) lacked PA data before or after the outcome of interest; (3) lacked any data for covariates; (4) had presented a recurrent event during the follow-up period, or (5) had presented a CVD event before the first follow-up.

### Matching

Each participant who presented with a first coronary event was matched with three controls devoid of coronary events and for whom subjective or objective PA was available at both follow-ups. Matching was performed in gender then on age, as these variables have been shown to influence PA levels [24, 25].

### Statistical analysis

Statistical analyses were performed using Stata version 16.1 for windows (Stata Corp, College Station, Texas, USA). Descriptive results were expressed as number of participants (percentage) for categorical variables and as average ± standard deviation or median [interquartile range] for continuous variables. The associations between the delay between the event and the second PA assessment and changes in PA were determined using Spearman nonparametric correlation.

For the paired analysis, paired comparisons (before-after) were performed using Wilcoxon’s signed rank test. For the matched analysis, comparisons between participants with and without the outcome of interest were performed by comparing changes in PA between the two periods, using mixed models taking into account the matching. Briefly, a variable indicative of case/control status was included as the fixed effect, while a variable indicating the pair (i.e., corresponding to one case and matched controls) was considered as the random effect. Statistical significance was assessed for a two-sided test with *p* < 0.05.

Power analyses were conducted to assess the sample size needed to detect minimal clinically meaningful difference of 10 min per day of moderate PA, using the corresponding variance as observed in this study. The statistical power of our sample size to detect such a difference was also computed. Calculations for both self-reported and accelerometer-assessed PA were performed. The following hypotheses were considered: paired sample, one-sided test, alpha level of 0.05, and for sample size a power of 80% and 90%.

## Results

### Characteristics of participants

Between the first and the second follow-up, 144 participants developed a first coronary event. Of those, 106 (73.6%) did not attend the second follow-up, and a further 1 (0.7%) did not provide PA data either at first or second follow-ups, leaving 37 (11.2%) participants for analysis. The characteristics of the included and excluded participants is summarized in supplementary Table 1. Excluded participants were more frequently current smokers and of lower educational level, and presented more frequently with obesity.

Between the second and the third follow-up, 74 participants developed a first coronary event. Of those, 13 (17.6%) did not attend the third follow-up, and 29 (39.2%) had no PA data either at second or third follow-ups, leaving 32 (43.2%) participants for analysis. The characteristics of the included and excluded participants is summarized in supplementary Table 1; no differences were found between included and excluded participants regarding all variables considered.

### Self-reported physical activity before and after a first coronary event

The results for the self-reported PA before and after the CHD event are summarized in Table 1. Participants spent most of their time in sedentary behaviours and only 2% in vigorous activities; no changes in PA levels were found before and after the first coronary event. No association was found between the time between the first coronary event and the second PA evaluation and changes in self-reported PA levels (supplementary Table 3).

**Table 1:** Physical activity levels before and after a cardiovascular event, CoLaus|PsyCoLaus study, Lausanne, Switzerland

	Before	After	P-value
**Self-reported time (min/day), ** ***N*** ** = 37**			
Sedentary	513 [450–635]	535 [465–642]	0.444
Light	151 [77–259]	166 [126–222]	0.380
Moderate	167 [104–250]	153 [109–240]	0.409
Vigorous	19 [0–48]	9 [0–31]	0.308
**Self-reported (% time)**			
Sedentary	55.7 [48.4–67.2]	60.5 [48.1–68.8]	0.690
Light	16.3 [9.1–27.8]	16.8 [14.8–23.1]	0.485
Moderate	18.7 [11.1–26.2]	16.3 [11.1–25.2]	0.325
Vigorous	2 [0–5.2]	0.9 [0–3.9]	0.321
**Accelerometer-assessed time (min/day), ** ***N*** ** = 32**			
Sedentary	750 [711 ; 808]	715 [688 ; 761]	0.048
Light	97 [81 ; 122]	87 [67 ; 111]	0.106
Moderate	18 [16 ; 28]	20 [11 ; 26]	0.184
Vigorous	1 [1 ; 3]	2 [1 ; 2]	0.567
**Accelerometer-assessed (% time)**			
Sedentary	86.6 [83.4 ; 89.1]	86.3 [84.2 ; 90.0]	0.564
Light	10.7 [8.5 ; 13.4]	10.6 [8.4 ; 12.7]	0.500
Moderate	2.2 [1.8 ; 3.1]	2.4 [1.4 ; 3.2]	0.421
Vigorous	0.1 [0.1 ; 0.3]	0.2 [0.1 ; 0.2]	0.599
**Walking metrics** (***N* = 30)**			
Steps / day	5961 [4734–8354]	5487 [4395–6760]	0.245
Mean cadence, steps / min	111 [108–112]	107 [106–109]	< 0.001
Mean walking bouts, sec	39 [37–42]	38 [35–42]	0.505

Objective physical activity data was processed using the GGIR software. Results are expressed as median [interquartile range] and analysis was conducted using Wilcoxon signed rank test.

Overall, 107 controls could be matched to 36 participants who developed a first coronary event and for whom self-reported PA was available. No differences were found regarding educational level between participants who developed a first coronary event and matched controls (not shown). The results between participants who developed a first coronary and gender- and age-matched controls are summarized in Table 2. Both groups reported an increase in time spent in sedentary behaviour, and a decrease in time spent in moderate PA; no differences were found between cases and controls regarding self-reported PA.

**Table 2:** Changes in physical activity levels in participants who developed a first coronary event and gender- and age-matched controls, CoLaus|PsyCoLaus study, Lausanne, Switzerland

	Controls (*N* = 107)	Cases (*N* = 36)	P-value
**Self-reported (min/day)**			
Sedentary	14 ± 15	21 ± 25	0.803
Light	-4 ± 11	12 ± 19	0.485
Moderate	-13 ± 13	-16 ± 20	0.889
Vigorous	-1 ± 1	-2 ± 2	0.782
**Self-reported (% time)**			
Sedentary	1.3 ± 1.6	1.8 ± 2.6	0.878
Light	-0.2 ± 1.2	0.9 ± 1.9	0.618
Moderate	-1.1 ± 1.4	1.7 ± 2.0	0.782
Vigorous	-0.2 ± 0.7	-1.0 ± 1.2	0.575
**Accelerometer-assessed (min/day)**	**Controls** (***N* = 93)**	**Cases** (***N* = 31)**	
Sedentary	17 ± 12	-21 ± 21	0.112
Light	-6 ± 3	-2 ± 4	0.482
Moderate	-21 ± 5	-7 ± 8	0.130
Vigorous	-1 ± 1	-2 ± 1	0.226
**Accelerometer-assessed (% time)**			
Sedentary	2.7 ± 0.6	0.1 ± 1.1	0.043
Light	-0.5 ± 0.3	0.2 ± 0.4	0.207
Moderate	-2.1 ± 0.5	-0.1 ± 0.9	0.042
Vigorous	-0.1 ± 0.1	-0.2 ± 0.1	0.319

Results expressed as average ± standard error. Between-group comparisons performed using mixed methods. A negative value indicates a decrease, a positive value indicates an increase from the first to the second follow-up (self-reported data) or from the second to the third follow-up (accelerometer-assessed data)

### Accelerometer-assessed physical activity before and after a first coronary event

The results for the accelerometer-assessed PA before and after the CHD event are summarized in Table 1. Participants spent over three quarters of their time in sedentary behaviours and almost none in vigorous activities; no changes in PA levels were found before and after the first coronary event. Using data from the GENEACTIV software led to similar results (supplementary Table 3). No association was found between the time between the first coronary event and the second PA evaluation and changes in accelerometer-assessed PA (supplementary Table 2).

For 31 out of the 32 cases, 93 gender- and age-matched controls were obtained. Their PA levels are indicated in supplementary Table 4. No differences were found regarding educational level between participants who developed a first coronary event and matched controls (not shown). The results between participants who developed a first coronary event and gender- and age-matched controls are summarized in Table 2. Controls had an increase in sedentary behaviour, while the opposite trend was found for cases; no differences were found between cases and controls regarding time spent in PA. When PA was reported as % of time, cases had a lower increase in sedentary behaviour and a lower decrease in moderate PA levels than controls (*p* = 0.05), while no differences were found for the other PA metrics.

Walking metrics were available for 30 of the 32 participants. Participants walked over 5,000 steps per day; no changes were found for most walking metrics, except for mean cadence, which decreased from 111 to 107 steps/min after the CHD event (Table 1).

### Power analyses

The results of the power analyses are summarized in supplementary Table 5. Briefly, to detect an increase in 10 min per day of moderate PA, the minimum sample size needed would be 584 for self-reported data and 122 for accelerometer-assessed data, using 80% power. The power of the current sample sizes to detect such a difference was 15% for the self-reported data and 35% for the accelerometer-assessed data (supplementary Table 5).

## Discussion

Our study suggests that patients who developed a first coronary event do not seem to significantly change their level of PA after a first coronary event, thus contradicting our initial hypothesis.

### Physical activity before and after a CHD event

In both paired and matched analyses, participants presenting with a first coronary event did not change their self-reported PA levels, while slight changes in objectively assessed PA were noted. Still, the differences in sedentary and moderate PA between cases and controls were small and due to a trend towards less PA among controls than to an increase in PA levels among cases. A recent study from Kang et al. [10] assessed the impact of a change in PA behaviours on mortality following a cardiovascular event. Patients who started exercising and those who kept on exercising showed a significantly reduced risk of all-cause mortality compared to patients who remained sedentary. The study also suggested that the potential benefits of regular PA beyond the age of 75 are lower than in younger subjects but are still significant. This reinforces the idea that PA after a cardio-vascular event is recommended at any age. Indeed, exercise-based cardiac rehabilitation has been proven to be an effective way of reducing CHD mortality and it can also lead to savings in healthcare resources if done correctly [1, 4]. A recent meta-analysis suggested that adherence rates can be as high as 77% and do not differ between clinic and home-based programs [26]. Still, in Switzerland, it is estimated that less than 50% of CHD patients participate in a cardiac rehabilitation program [27], although many specialized centres exist throughout the country and cardiac rehabilitation programs can be covered by the basic health insurance on medical prescription. As we did not collect information regarding rehabilitation, we could not infer the effect of the latter on PA levels. Overall, our results suggest that people who present with a first coronary event fail to increase their PA after the event.

Walking metrics did not change before and after the coronary event, except for a decrease in maximal cadence. We failed to find any study assessing walking metrics before and after a coronary event; hence, comparison with the literature was not possible. Several studies have shown that slow gait speed is significantly associated with an increased risk of cardiovascular events [28, 29]. Our results thus suggest that our participants should maintain or even increase their gait speed and/or number of steps after a CHD event.

### Implications for clinical practice

The patients health competence to understand the need to maintain or increase their PA, and strategies for developing self-efficacy, can be implemented [30]. As functional improvement after cardiac rehabilitation is mostly related to non-cardiac factors such as age, gender and corpulence [31], one way to improve PA levels of patients who presented a coronary event would be to promote resilience resources such as sense of coherence [32]. Other options include raising awareness of cardiovascular risk factors [33] and maintaining a good communication with patients, as their interest in health information tends to decrease with time [34]. Finally, although PA has been highlighted as a factor regarding the prevention of CVD [35], the absence of information for the patients and health providers to guide them through the healthcare system and/or refer them to existing PA measures and providers is a major barrier towards the implementation of PA.

### Strengths and limitations

To our knowledge, this is one of the few studies assessing physical activity before and after a CHD event, using both self-reported and accelerometer-assessed methods. We used two different software to extract PA metrics from the accelerometer files, and results were similar. We also compared participants who presented with a first coronary event with gender- and age-matched healthy controls. According to our results, the healthy controls do not seem to change their physical activity either. This reinforces the idea that presenting a coronary event does not lead to changes in PA levels following the event.

This study also has several limitations. Firstly, the number of participants was too small to obtain significant results. The main reason for the limited sample size was the difficulty of repeatedly obtaining data of sufficiently good quality to be analysed in all patients: some patients quit the study; others did not wear the accelerometer or did not answer the questionnaires as planned, making their data unusable. Still, despite the high attrition rate, included and excluded participants were rather comparable, mainly for the objective PA data. A previous study conducted in Korea also reported a high exclusion rate, as from the initial 78’533 patients with newly diagnosed CVD, only 6076 (7.7%) had PA data for analysis [10]. It would therefore be interesting to replicate our study with a larger sample size or to aggregate the findings of several studies in a meta-analysis. Secondly, the analysis was carried out mainly on the French-speaking Swiss population and is therefore not necessarily applicable to the country as a whole. Indeed, the majority of out- and in-patient cardiac rehabilitation programs are located in the German-speaking part of Switzerland [27], which could preclude our participants to attend a cardiac rehabilitation. Thirdly, the results of the accelerometer-assessed PA differed considerably according to the software applied. Indeed, in a previous study, the percentage of participants complying with the PA recommendations varied from 50.2 to 99.8% depending on the threshold, the software or version of the software used [36]. Overall, it would be important that future studies provide the software and the thresholds used to define the intensity of PA to allow comparison with other studies [37]. Still, the conclusions were identical irrespective of the software or the thresholds applied. Finally, we could not adjust for several factors differing between cases and controls such as number of comorbidities, number of drugs, living arrangement or number of physician consultations. Hence, the issue of residual confounding cannot be ignored.

We conclude that patients do not seem to change their PA levels after a first coronary event. Our results should be confirmed in larger samples.

### Electronic supplementary material

Below is the link to the electronic supplementary material.

Supplementary Material 1: Information 1. Example of calculation of standardized physical activity as percentage of non-sleep time, 2. Clinical data collection in the CoLaus Study 3. Supplementary tables 1-5

## Data Availability

The CoLaus|PsyCoLaus cohort data used in this study cannot be fully shared as they contain potentially sensitive patient information. As discussed with the competent authority, the Research Ethic Committee of the Canton of Vaud, transferring or directly sharing this data would be a violation of the Swiss legislation aiming to protect the personal rights of participants. Non-identifiable, individual-level data are available for interested researchers, who meet the criteria for access to confidential data sharing, from the CoLaus Datacenter (CHUV, Lausanne, Switzerland). Instructions for gaining access to the CoLaus data used in this study are available at https://www.colaus-psycolaus.ch/professionals/how-to-collaborate/.
