# Supplementary material for: It needs more than a myocardial infarction to start exercising: the CoLaus|PsyCoLaus prospective study
Source: BMC Cardiovasc Disord. 2024 Feb 12;24:102. doi: 10.1186/s12872-024-03755-9 (PMC10863136; doi:10.1186/s12872-024-03755-9)
Supplement: Supplementary file 1 — Supplementary Material 1: Information 1. Example of calculation of standardized physical activity as percentage of non-sleep time, 2. Clinical data collection in the CoLaus Study 3. Supplementary tables 1-5 [file 12872_2024_3755_MOESM1_ESM.docx]

**Supplementary information**

1. **Example of calculation of standardized physical activity as percentage of non-sleep time**

Participant reporting 510 minutes of sleep, 600 minutes of sedentary, 282 minutes of light, 167 minutes of moderate and 86 minutes of vigorous physical activity.

Reported time spent in non-sleeping activities: 600+282+167+0=1049 minutes

Estimated time spent in non-sleeping activities: (NST) 1440-510=840 minutes

Standardized non-sleeping time spent in

$$Sedentary=\frac{600}{1049}\times840=480 minutes/day$$

$$Light=\frac{282}{1049}\times840=226 minutes/day$$

$$Moderate=\frac{86}{1049}\times840=69 minutes/day$$

$$Vigorous=\frac{0}{1049}\times840=0 minutes/day$$

The standardized non-sleeping times were used in the computations.

1. **Clinical data collection in the CoLaus Study**

Prevalent and incident CV events were recorded through a stepwise process.

- First, relevant medical records of participants who declared, during the baseline and/or follow-up examinations, to have presented a CVD and/or CVD-related procedure during their lifetime, including MI, angina pectoris, stroke, arrhythmia, cardiomyopathy, coronarography and/or percutaneous transluminal coronary angioplasty (PTCA) and/or coronary stenting, coronary artery bypass grafting (CABG) and peripheral artery disease (PAD), were collected. The records were collected from general practitioners, cardiologists, neurologists and/or hospitals (as appropriate), and encompassed medical and/or surgical notes, laboratory, radiological, echocardiographic and electrocardiographic reports. If necessary, the original coronarography (angiogram) and brain CT/MRI exams were collected.
- Second, to retrieve events that may not have been mentioned during interviews, the central medical database of the University Hospital of Lausanne, which is the main community hospital in the catchment area of the study, was searched. Participants with hospital records were identified by cross-checking with administrative data and events of interest were detected using the following ICD-10 (*International Classification of diseases, Tenth Edition*) codes: I20.0, I21.-, I22.-, I24.-, I25.1-, I25.2-, I25.5, I25.6, I25.8, I25.9, I61.-, I62.-, I63.-, I64, I69.1, I69.2, I69.3, I69.4, I69.8, and G45.-.

1. **Adjudication of coronary events**

All coronary-related events were adjudicated by two cardiologists based upon an international expert consensus document.[1] For this study, we defined major coronary events as a composite outcome including: i) acute coronary syndromes (ACS) (acute myocardial infarction (AMI) or unstable angina) and ii) symptomatic stable angina followed by a revascularization procedure, either by percutaneous coronary intervention (PCI) or by coronary artery bypass grafting (CABG). Iatrogenic events caused by a medical procedure (angioplasty, stenting or coronary artery bypass surgery) were not considered. The following criteria were applied:

1. Definite AMI, including ST-segment elevation myocardial infarction (STEMI) and Non-ST-segment elevation myocardial infarction and (NSTEMI) was defined in the presence of at least one of the following criteria:
2. Detection of rise/fall of Troponin I or T with at least one value above the 99^th^ percentile of the upper reference limit together with evidence of myocardial ischaemia with *at least one* of the following:
   - Symptoms of ischaemia
   - ECG changes indicative of new ischaemia (new ST-T changes or new left bundle branch block)
   - Development of pathological Q waves in the ECG
   - Echocardiographic evidence of new segmental abnormality
3. Sudden, unexpected cardiac death, involving cardiac arrest, often with symptoms suggestive of myocardial ischaemia, and accompanied by presumably new ST elevation, or new left bundle branch block

**and/or**

- - Evidence of fresh thrombus by coronary angiography and/or at autopsy

**but**

- - Occurring before blood samples could be obtained, or at a time before the appearance of cardiac biomarkers in the blood (Troponin I or T).

1. Due to missing information on cardiac biomarkers, probable AMI was defined using the following criteria:
2. Symptoms of ischaemia

**and**

1. ECG changes: new ST-T changes, new left bundle branch block or new pathological Q waves

**and/or**

1. Coronary angiography followed by percutaneous or surgical revascularization

**and/or**

1. Echocardiographic evidence of new segmental abnormality
2. Unstable angina was defined using the following criteria (used by Cardiobase/SPUM)**:**
3. Symptoms of ischaemia
4. Hospitalization or ambulatory care
5. Negative troponin values
6. Change in medication after hospital discharge or during ambulatory care (betablocker, calcium blocker, nitro-derivatives, molsidomine, nicorandil)
7. Coronary artery disease (CAD) events corresponded to participants who presented with typical symptoms (stable angina) and underwent either percutaneous (PTCA ± stenting) or surgical (CABG) revascularizations, unless these procedures were directly related to an AMI. When the exact date of the event could not be traced, the date of the revascularization was used (n=4). The following criteria were used to define CAD:
8. Symptoms of ischaemia

**and**

1. Coronary angiography followed by percutaneous or surgical revascularization

**without:**

- - Acute context
  - ECG changes compatible with AMI
  - Modification in medication (-> unstable angina).

1. Detection of rise/fall of troponin I or T

**Reference**

1. Thygesen, K., et al., *Universal definition of myocardial infarction.* Eur Heart J, 2007. **28**(20): p. 2525-38.

**Supplementary table 1**: clinical characteristics of the participants who presented a first coronary event during follow-up, according to exclusion criteria and type of physical activity assessment, CoLaus|PsyCoLaus study, Lausanne, Switzerland

|  |  | **Subjective** |  |  | **Objective** |  |
| --- | --- | --- | --- | --- | --- | --- |
|  | **Included** | **Excluded** | **P-value** | **Included** | **Excluded** | **P-value** |
| Sample size | **37** | **107** |  | **32** | **42** |  |
| Age (years) | 64.3 ± 9.4 | 65.6 ± 10.0 | 0.494 | 66.2 ± 8.7 | 69.5 ± 9.2 | 0.124 |
| Women (%) | 10 (27.0) | 37 (34.6) | 0.398 | 16 (50.0) | 18 (42.9) | 0.541 |
| Body mass index (kg/m^2^) | 26.2 ± 3.5 | 28.3 ± 4.9 | 0.016 | 27.8 ± 4.5 | 28.0 ± 5.2 | 0.900 |
| BMI categories (%) |  |  | 0.013 |  |  | 0.321 |
| Normal | 14 (37.8) | 26 (25.0) |  | 9 (28.1) | 12 (30.0) |  |
| Overweight | 20 (54.1) | 44 (42.3) |  | 17 (53.1) | 15 (37.5) |  |
| Obese | 3 (8.1) | 34 (32.7) |  | 6 (18.8) | 13 (32.5) |  |
| Education level (%) |  |  | 0.018 |  |  | 0.860 |
| High | 17 (46.0) | 24 (22.4) |  | 6 (18.8) | 10 (23.8) |  |
| Middle | 13 (35.1) | 45 (42.1) |  | 7 (21.9) | 8 (19.1) |  |
| Low | 7 (18.9) | 38 (35.5) |  | 19 (59.4) | 24 (57.1) |  |
| Smoking categories (%) |  |  | 0.018 |  |  | 0.478 |
| Never | 17 (46.0) | 24 (22.4) |  | 5 (17.2) | 12 (30) |  |
| Former | 13 (35.1) | 45 (42.1) |  | 13 (44.8) | 15 (37.5) |  |
| Current | 7 (18.9) | 38 (35.5) |  | 11 (37.9) | 13 (32.5) |  |
| Blood pressure (mm Hg) |  |  |  |  |  |  |
| Systolic | 138 ± 20 | 134 ± 16 | 0.212 | 133 ± 20 | 134 ± 19 | 0.859 |
| Diastolic | 80 ± 12 | 79 ± 11 | 0.899 | 81 ± 10 | 77 ± 11 | 0.193 |
| Hypertension (%) | 25 (67.6) | 66 (62.3) | 0.564 | 17 (53.1) | 30 (71.4) | 0.105 |
| Diabetes (%) | 8 (21.6) | 27 (25.2) | 0.659 | 6 (18.8) | 9 (22.0) | 0.737 |

Results are expressed as mean ± standard deviation or as number of participants (percentage). Between-group comparison performed using student’s t-test for continuous variables and chi-square test for categorical variables.

**Supplementary table 2**: association between time between the first coronary event and the second physical activity assessment and changes in physical activity levels, CoLaus|PsyCoLaus study, Lausanne, Switzerland.

|  | **Subjective PA** | **Objective PA** |
| --- | --- | --- |
| Absolute values |  |  |
| Sedentary | -0.112 | -0.049 |
| Light | -0.067 | 0.256 |
| Moderate | 0.224 | 0.148 |
| Vigorous | -0.018 | 0.175 |
| As percentage of time |  |  |
| Sedentary | -0.059 | -0.150 |
| Light | -0.050 | 0.324 |
| Moderate | 0.214 | 0.016 |
| Vigorous | -0.008 | 0.103 |

Results are expressed as Spearman rank correlation coefficients. All associations are nonsignificant at p>0.05

**Supplementary table 3**: objectively assessed physical activity levels before and after a first coronary event, CoLaus|PsyCoLaus study, Lausanne, Switzerland.

|  | Before | After | P-value |
| --- | --- | --- | --- |
| Time (min/day) |  |  |  |
| Sedentary | 610 [545 - 659] | 602 [540 - 624] | 0.573 |
| Light | 95.8 [79 - 113] | 95.9 [79 - 117] | 0.993 |
| Moderate | 159 [113 - 189] | 141 [111 - 189] | 0.319 |
| Vigorous | 1 [1 - 4] | 1 [0 - 3] | 0.237 |
| Percentage of daily time |  |  |  |
| Sedentary | 70.4 [66.8 - 76.9] | 71.2 [66.2 - 73.5] | 0.978 |
| Light | 11.0 [9.6 - 13.5] | 11.2 [9.6 - 14.2] | 0.638 |
| Moderate | 17.4 [14.4 - 21.3] | 17.6 [14.5 - 22.0] | 0.561 |
| Vigorous | 0.1 [0.1 - 0.4] | 0.2 [0 - 0.3] | 0.304 |

Data for 32 participants. Physical activity data processed using the GENEACTIV software. Results expressed as median [interquartile range]. Paired analysis using Wilcoxon signed rank test.

**Supplementary table 4**: changes in physical activity levels in participants who developed a first coronary event and gender- and age-matched controls, CoLaus|PsyCoLaus study, Lausanne, Switzerland.

|  | Controls (N=107) | Cases (N=36) | P-value |
| --- | --- | --- | --- |
| Self-reported (min/day) |  |  |  |
| Sedentary | 20 [-39 ; 102] | 11 [-69 ; 105] | 0.556 |
| Light | -2 [-62 ; 48] | 19 [-52 ; 105] | 0.194 |
| Moderate | -7 [-87 ; 47] | -6 [-67 ; 41] | 0.987 |
| Vigorous | 0 [-12 ; 18] | 0 [-24 ; 5] | 0.203 |
| Self-reported (% time) |  |  |  |
| Sedentary | 3.0 [-4.1 ; 11.7] | 1.3 [-8.9 ; 10.8] | 0.428 |
| Light | 0.1 [-6.8 ; 5.0] | 1.7 [-5.7 ; 10.5] | 0.264 |
| Moderate | -1.1 [-10.2 ; 5.3] | -1.5 [-6.6 ; 3.9] | 0.957 |
| Vigorous | 0 [-1.3 ; 1.9] | 0 [-2.6 ; 0.6] | 0.197 |
| Accelerometer-assessed (min/day) | **Controls (N=93)** | **Cases (N=31)** |  |
| Sedentary | 12 [-34 ; 58] | -13 [-65 ; 50] | 0.242 |
| Light | -8 [-24 ; 8] | -2 [-19 ; 16] | 0.236 |
| Moderate | -19 [-46 ; 2] | -10 [-32 ; 20] | 0.161 |
| Vigorous | -0.2 [-1 ; 0.3] | -0.2 [-0.9 ; 0.4] | 0.935 |
| Accelerometer-assessed (% time) |  |  |  |
| Sedentary | 2.7 [-0.9 ; 5.9] | 0.4 [-2.8 ; 3] | 0.039 |
| Light | -0.8 [-2.3 ; 1.1] | 0 [-1.5 ; 1.6] | 0.148 |
| Moderate | -2.4 [-4.2 ; 1.0] | -0.3 [-3.1 ; 2.3] | 0.058 |
| Vigorous | 0 [-0.1 ; 0.1] | 0 [-0.1 ; 0.1] | 0.869 |

Results expressed as median [interquartile range]. A negative value indicates a decrease, a positive value indicates an increase from the first to the second follow-up (self-reported data) or from the second to the third follow-up (accelerometer-assessed data). Between-group comparisons performed using Kruskal-Walllis test.

**Supplementary table 5**: sample size needed to detect an increase of 10 minutes per day in moderate physical activity, using data from self-reported and accelerometer-assessed physical activity.

|  | SD used | Power of 80% | Power of 90% |
| --- | --- | --- | --- |
| Sample size |  |  |  |
| Self-reported | 97 | 584 | 808 |
| Accelerometer-assessed | 44 | 122 | 168 |
| Power | **SD used** | **Sample size** | **Power** |
| Self-reported | 97 | 37 | 15% |
| Accelerometer-assessed | 44 | 32 | 35% |

SD, standard deviation of the difference in moderate physical activity between first and second evaluation, for participants who presented with a first coronary event. Calculations performed using the following hypotheses: paired sample, one-sided test, alpha level of 0.05.
